# Investigation of Bandgap Properties of a Piezoelectric Phononic Crystal Plate Based on the PDE Module in COMSOL

**DOI:** 10.3390/ma17102329

**Published:** 2024-05-14

**Authors:** Guoqing Liu, Denghui Qian

**Affiliations:** School of Naval Architecture & Ocean Engineering, Jiangsu University of Science and Technology, Zhenjiang 212100, China

**Keywords:** piezoelectric phononic crystal plate, partial differential equation, band structure, vibration mode, transmission curve

## Abstract

Aiming to address the vibration noise problems on ships, we constructed a piezoelectric phononic crystal (PC) plate structure model, solved the governing equations of the structure using the partial differential equations module (PDE) in the finite element softwareCOMSOL6.1, and obtained the corresponding energy band structure, transmission curves, and vibration modal diagrams. The application of this method to probe the structural properties of two-dimensional piezoelectric PCs is described in detail. The calculation results obtained using this method were compared with the structures obtained using the traditional plane wave expansion method (PWE) and the finite element method (FE). The results were found to be in perfect agreement, which verified the feasibility of this method. To safely and effectively adjust the bandgap within a reasonable voltage range, this paper explored the order of magnitude of the plate thickness, the influence of the voltage on the bandgap, and the dependence between them. It was found that the smaller the order of magnitude of the plate thickness, the smaller the order of magnitude of the band in which the bandgap was located. The magnitude of the driving voltage that made the bandgap change became smaller accordingly. The new idea of attaching the PC plate to the conventional plate structure to achieve a vibration damping effect is also briefly introduced. Finally, the effects of lattice constant, plate width, and thickness on the bandgap were investigated.

## 1. Introduction

As ships become larger and lighter, the prevalent use of high-strength steel in hull structures reduces the hull structural stiffness to a certain extent. As the power of the main engine and propulsion unit increases, the excitation force of these devices is also increased, and ship vibration problems are increasingly exacerbated [1]. Once vibration reaches a critical point or resonance occurs [2,3], the hull structure is susceptible to damage due to fatigue. It will not only reduce the use, precision, and service life of the equipment and instruments on board ships, but also bring inconvenience to the regular work and life of the staff on the ship. Therefore, suppressing vibration caused by mechanical equipment on board ships and controlling the propagation of elastic waves are of great significance to the safety and comfort of staff on ships.

To effectively mitigate vibration problems on board ships, a great deal of research has been conducted by relevant scholars, proposed initially as flexible vibration isolation [4], i.e., elastic support between the equipment and the foundation. However, with the rapid development of the marine sector, the demand for vibration damping is also increasing. Conventional theoretical designs often struggle to achieve the requirements of low added mass, vibration isolation, and low sound isolation frequencies. Later, it was found that the periodic structure has bandgap properties [5,6,7,8], and elastic waves are blocked in the bandgap range. Consequently, new composites with periodic structures, such as PC [9,10], have received much attention. Guo et al. [11] proposed a PC plate structure with an additional cylindrical vibrator. Through the reasonable geometric parameter design and arrangement form, the structure of the low-frequency vibration isolation, in the field of ship vibration and noise, has very good application prospects. Yang and his co-workers [12] studied the low-frequency acoustic isolation characteristics of thin-film PCs to control low-frequency noise on ships. They explored the influence of the configuration of PCs on their acoustic isolation performance. Zhang and his co-workers [13] proposed a two-dimensional cylindrical periodic arrangement of PC plates and conducted simulation experiments using finite element software COMSOL. It has been proven that the PC plate can be used as vibration isolation material for the vibration-damping foot of the ship engine and can achieve a better vibration-damping effect. Ruan [14] addressed the low-frequency mechanical vibration of ship power systems by desiging and analyzing a single-phase helical PC based on local resonance modes and by conducting several sets of experimental tests to provide a possibility of practical application in ship vibration and noise reduction.

With the increasing demand for vibration and noise reduction in marine engineering applications, it is often necessary for structures to be able to change accordingly to environmental changes. Once a conventional PC structure is defined, its bandgap position and width cannot be changed. To better adapt to engineering needs, much research has been conducted on intelligent adjustable PCs [15,16] and elastic wave metamaterials [17,18]. In this regard, piezoelectric materials have critical applications in intelligent modulation [19]. Relevant scholars have combined piezoelectric materials with other kinds of materials to form piezoelectric PC structures [20], have achieved regulation of the bandgap by applying pre-stress [21], applied voltage [22], and external circuits [23], and have studied a variety of different methods to solve the corresponding structures. Chen et al. [24] periodically glued a piezoelectric layer on epoxy resin to form a piezoelectric PC structure, and the bandgap characteristics of the piezoelectric PC beams were calculated using the improved transfer matrix method (TM). Wang et al. [25] used a PWE method, based on the Mindlin piezoelectric plate theory, to derive the dispersion relation of bending waves in a non-uniform piezoelectric plate. Miao and his co-workers [26] implemented the derivation of the formulae for the energy band structures based on the finite element method and the plane wave expansion method. They obtained the energy band diagrams of the corresponding piezoelectric structures. Liao [27] proposed a new piezoelectric PC plate structure and obtained the corresponding energy band diagram and transmission curve based on COMSOL using the finite element method.

Most scholars have conducted theoretical studies on piezoelectric PC structures by improving the method of calculating conventional PC energy band structures, but the calculation methods have some limitations. For example, the traditional PWE method [28] is slow to converge when there are significant differences in the material parameters of the group elements, and it cannot obtain the transmission curve and displacement field diagram of piezoelectric PC structures simply and efficiently. When the PDE module in COMSOL is used to solve the PC structure, the computation time is long and inefficient. The TM method [29] is more suitable for 1D PC structures and cannot directly deal with 2D and 3D problems. The finite difference in the time domain method [30] is particularly time-consuming and inefficient for calculating 3D structures. Moreover, most of the research only focuses on the theoretical aspect. It does not propose practical application scenarios, and the application of piezoelectric materials in the marine field has not been extended.

This paper explores a model of a piezoelectric PC plate for marine applications. The governing equations of the structure were solved using the partial differential equations (PDE) module of the finite element software COMSOL6.1 to obtain the corresponding energy band structure, transmission curves, and vibration modal diagrams. To transform the governing equations of the piezoelectric PC structure into the form of the built-in equations in COMSOL6.1, a new set of reduced-order methods is proposed. The simulation results were compared with the calculation results of the traditional PC solution method to verify the feasibility and scientific validity of the method. To better meet the needs of practical engineering applications, we explored the order of magnitude of the plate thickness, the effect of voltage on the bandgap, and the dependence between them.

## 2. Modelling and Methodology

### 2.1. Construction of the Model

**Figure 1:**
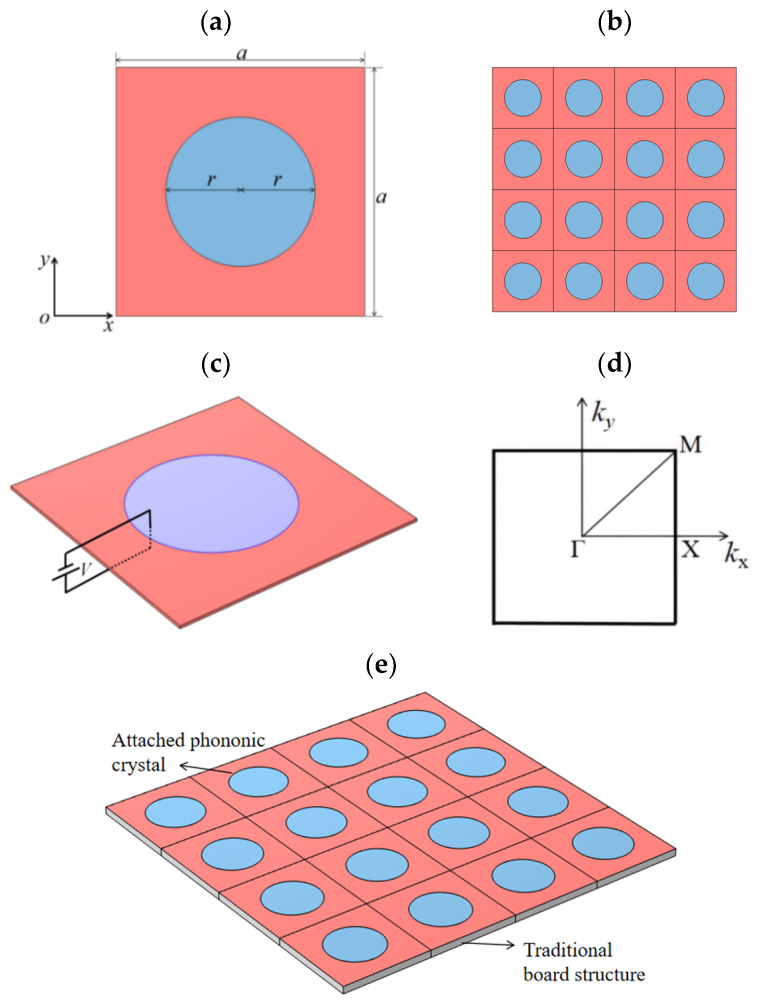
Structure diagrams: (**a**) schematic diagram of a single cell, (**b**) finite periodic sequence diagram, (**c**) applied voltage schematic, (**d**) the first Brillouin zone for the square lattice, and (**e**) attached phononic crystals.

A single cell of a piezoelectric PC plate structure is shown in Figure 1a. It consists of epoxy resin (the red area) and piezoelectric material PZT-4 (the blue area). The single-cell structure shown in Figure 1a is arranged in a square periodic arrangement along the *xy* plane, forming a piezoelectric PC plate (Figure 1b). The applied electric field on PZT-4 is denoted by *V*, the lattice constant is denoted by *a*, the radius of the built-in circle is denoted by *r*, and the thickness is denoted by *h*. Interfacial effects between the materials were not considered.

The geometric parameters above are *a* = 0.1 m and thickness *h* = 1 × 10^−4^ m. Considering that it is simpler and more convenient to apply small voltages in practical applications, we discussed the case where the applied voltage is −10 V.

As shown in Figure 1e, we proposed a new engineering application of piezoelectric PC structures attached to conventional plate structures to achieve vibration and noise reduction. In addition, the structure can also be attached directly to vibration-prone devices, such as engines or cavities.

### 2.2. Method of Obtaining the Energy Band Structure Diagram Based on the PDE Module

Based on the literature [31] and a series of formulae derivations, the control equations for the piezoelectric PC plate structure can be written uniformly in the following form:

(1) $$\frac{\partial^{2}}{\partial x^{2}}\left(m\frac{\partial^{2}W\left(x,y\right)}{\partial x^{2}}\right)+\frac{\partial^{2}}{\partial x^{2}}\left(n\frac{\partial^{2}W\left(x,y\right)}{\partial y^{2}}\right)+\frac{\partial^{2}}{\partial y^{2}}\left(n\frac{\partial^{2}W\left(x,y\right)}{\partial x^{2}}\right)+\frac{\partial^{2}}{\partial y^{2}}\left(m\frac{\partial^{2}W\left(x,y\right)}{\partial y^{2}}\right)+\;2\frac{\partial^{2}}{\partial x\partial y}\left(p\frac{\partial^{2}W\left(x,y\right)}{\partial x\partial y}\right)-\frac{\partial }{\partial x}\left(q\frac{\partial W\left(x,y\right)}{\partial x}\right)-\frac{\partial }{\partial y}\left(q\frac{\partial W\left(x,y\right)}{\partial y}\right)=\omega^{2}\cdot l\cdot W\left(x,y\right)$$

In the above equation, m, n, p, q, and l  are constants and ω denotes the frequency. The specific calculation formula is shown in Table 1, and the material parameters involved are shown in Table 2.

The PDE module in the finite element software COMSOL6.1 was used to solve Equation (1), which yielded the corresponding structure’s energy band diagram. The equation form chosen was the time-domain equation, and the following equation represents the form of the built-in time-domain equation in COMSOL6.1:

(2) $$e_{a}\frac{\partial^{2}u}{\partial t^{2}}-d_{a}\frac{\partial u}{\partial y}+\nabla \cdot \Gamma =f$$

where *u* is the variable to be solved, and *e_a_*, *d_a_*, *f*, and Γ represent the mass coefficient, the damped mass coefficient, the source term, and the conserved flux, respectively. Damping was not considered since the highest order of the built-in equation form in COMSOL6.1 was 2. COMSOL6.1 can only solve 2nd-order equations [32], so we needed to reduce Equation (1) to a lower order. In addition, to simulate the periodicity of the structure, we needed to impose the periodicity condition in COMSOL6.1. The periodicity type chosen was continuity.

Based on the periodic displacement field of the structure, $W\left(x,y\right)$ in Equation (1) can be decomposed, according to Bloch’s theorem, as:

(3) $$W\left(x,y\right)=e^{i(k_{x}x+k_{y}y-\omega t)}\cdot W_{K}$$

*k_x_* and *k_y_* represent the wavevectors restricted to the first Brillouin zone, *t* represents time, $W_{K}$ represents a function with the same period as each material parameter, and the other items are similar. It is worth noting that Equation (3) was also the expression used to obtain the corresponding modal displacement field diagrams.

The second-order derivatives of Equation (3) are obtained for *x* and *y*, respectively:

(4) $$\frac{\partial^{2}W}{\partial x^{2}}=-k_{x}^{2}e^{i\left(k_{x}x+k_{y}y-\omega t\right)}W_{K}+ik_{x}e^{i\left(k_{x}x+k_{y}y-\omega t\right)}W_{Kx}+ik_{x}e^{i\left(k_{x}x+k_{y}y-\omega t\right)}W_{Kx}+e^{i\left(k_{x}x+k_{y}y-\omega t\right)}W_{Kxx}$$

(5) $$\frac{\partial^{2}W}{\partial y^{2}}=-k_{y}^{2}e^{i\left(k_{x}x+k_{y}y-\omega t\right)}W_{K}+ik_{y}e^{i\left(k_{x}x+k_{y}y-\omega t\right)}W_{Ky}+ik_{y}e^{i\left(k_{x}x+k_{y}y-\omega t\right)}W_{Ky}+e^{i\left(k_{x}x+k_{y}y-\omega t\right)}W_{Kyy}$$

$W_{Kx}$ and $W_{Kxx}$ denote the first- and second-order partial derivatives of $W_{K}$, respectively, with respect to *x*. The other items are similar.

Due to the periodicity of the structure, the material composition of m, n, p, q, and l is also a periodic function in space, for which partial derivatives need to be considered. For this reason we introduced five dependent variables for the order reduction, as follows:

(6) $$W_{1}=m\frac{\partial^{2}W\left(x,y\right)}{\partial x^{2}}\;\;\;W_{2}=n\frac{\partial^{2}W\left(x,y\right)}{\partial y^{2}}\;\;\;W_{3}=n\frac{\partial^{2}W\left(x,y\right)}{\partial x^{2}}\;W_{4}=m\frac{\partial^{2}W\left(x,y\right)}{\partial y^{2}}\;\;\;W_{5}=p\frac{\partial^{2}W\left(x,y\right)}{\partial x\partial y}$$

$W_{1}$ can also be decomposed into the following form according to Bloch’s theorem:

(7) $$W_{1}=m\frac{\partial^{2}W\left(x,y\right)}{\partial x^{2}}=e^{i(k_{x}x+k_{y}y-\omega t)}\cdot W_{1K}$$

Substituting Equation (4) in and combining it with Equation (7) yields the following:

(8) $$W_{1K}=m\left(-{k_{x}}^{2}W_{K}+ik_{x}W_{Kx}+ik_{x}W_{K}+W_{Kxx}\right)$$

Note that since both $W_{1}$ and W contain $e^{i(k_{x}x+k_{y}y)}$, the relationship between $W_{1}$ an W is equivalent to the relationship between $W_{1K}$ and $W_{K}$. Similarly, the following relationships between $W_{2\sim 5}$ and W were obtained:

(9) $$W_{2K}=n\left(-{k_{y}}^{2}W_{K}+ik_{y}W_{Ky}+ik_{y}W_{K}+W_{Kyy}\right)$$

(10) $$W_{3K}=n\left(-{k_{x}}^{2}W_{K}+ik_{x}W_{Kx}+ik_{x}W_{K}+W_{Kxx}\right)$$

(11) $$W_{4K}=m\left(-{k_{x}}^{2}W_{K}+ik_{x}W_{Kx}+ik_{x}W_{K}+W_{Kxx}\right)$$

(12) $$W_{5K}=p\left(-k_{x}{k_{y}W}_{K}+ik_{x}W_{Ky}+ik_{y}W_{Kx}+W_{Kxy}\right)$$

At this point, Equation (1) can be successfully converted to a second-order equation. The final form of the solution of Equation (1) in COMSOL6.1 is shown in Equation (13) (i.e., the form of input). It is worth noting that when solving Equation (13) in COMSOL6.1’s PDE module, the order of partial derivatives can have a significant impact on the results. Therefore, it is essential to set the parameters correctly. In Equation (13), the left part of the equal sign is incorporated into the source term *f* and the conserved flux Γ. Because of the similarity of these terms and the same principle of implementation in COMSOL6.1, we only used the first term as an example in this paper and incorporated the right side of the equal sign into the quality factor *e*_a_. Table 3 details how to set each parameter in COMSOL6.1.

In addition to this, we needed to establish the relationship between $W_{1\sim 5}$ (i.e., $W_{1}$ to $W_{5}$) and W in COMSOL6.1 (by entering Equations (8)–(12) in COMSOL6.1’s physical field). Table 4 shows the setting of each parameter, for example, $W_{1}$ (i.e., the realization of Equation (8)).

(13) $$\begin{array}{ll} (-k_{x}^{2}W_{1K}+ik_{x}W_{1Kx} & +ik_{x}W_{1Kx}+W_{1Kxx})+\left(-k_{x}^{2}W_{2K}+ik_{x}W_{2Kx}+ik_{x}W_{2Kx}+W_{2Kxx}\right)\\ & +\left(-k_{y}^{2}W_{3K}+i{k_{y}W}_{3Ky}+ik_{y}W_{3Ky}+W_{3Kyy}\right)\\ & +\left(-k_{y}^{2}W_{4K}+ik_{y}W_{4Ky}+ik_{y}W_{4Ky}+W_{4Kyy}\right)\\ & +2\left(-k_{x}k_{y}W_{5K}+ik_{x}W_{5Ky}+ik_{y}W_{5Kx}+W_{5Kxy}\right)\\ & -q\left(-k_{x}^{2}W_{K}+ik_{x}W_{Kx}+ik_{x}W_{Kx}+W_{Kxx}\right)\\ & -\left(-k_{y}^{2}W_{K}+ik_{y}W_{Ky}+ik_{y}W_{Ky}+W_{Kyy}\right)=-\omega^{2}\cdot l\cdot W_{K} \end{array}$$

$\tilde{c_{11}}=c_{11}-c_{13}^{2}/c_{33}$, $\tilde{c_{12}}=c_{12}-c_{13}^{2}/c_{33}$, $\tilde{e_{31}}=e_{31}-c_{13}e_{33}/c_{33}$, $\tilde{c_{66}}=c_{66}$, and $\tilde{\kappa_{33}}{=\kappa }_{33}{+e}_{33}^{2}/c_{33}$ were included among these.

### 2.3. Method of Obtaining the Transmission Curve Diagram Based on the PDE Module

We also solved Equation (1) through the PDE module in COMSOL6.1 and then obtained the transmission curve diagram of the finite periodic sequence structure. At this point, the form of the COMSOL6.1 built-in equations was changed to the study control. Assuming that the frequency domain equations were chosen for the equations, Equation (14) was as follows:

(14) $$-e_{a}\omega^{2}u-d_{a}i\omega u+\nabla \cdot \Gamma =f$$

Since the order of the equations in Equation (10) did not exceed order 2, we still needed to perform an order reduction of Equation (1). It is worth noting that the reduced-order method used in the first half of this article to obtain the energy band structure diagram was only applicable to infinite-period structures and is no longer suitable for obtaining the transmission curve for finite-period sequence structures. For this reason, we proposed a new reduced-order method.

Here, we also introduce the five dependent variables illustrated in Equation (6). Instead of expanding them in the form of derivatives, we substituted them directly into Equation (1), which was transformed into the following form:

(15) $$\frac{\partial^{2}W_{1}}{\partial x^{2}}+\frac{\partial^{2}W_{2}}{\partial x^{2}}+\frac{\partial^{2}W_{3}}{\partial y^{2}}+\frac{\partial^{2}W_{4}}{\partial y^{2}}+2\frac{\partial^{2}W_{5}}{\partial x\partial y}-\frac{\partial }{\partial x}\left(q\frac{\partial W\left(x,y\right)}{\partial x}\right)\;-\frac{\partial }{\partial y}\left(q\frac{\partial W\left(x,y\right)}{\partial y}\right)=\omega^{2}\cdot l\cdot W\left(x,y\right)$$

At this point, the entire left side of the equal sign in Equation (15) was put into the conserved flux Γ. The source term *f* and the mass damping coefficient *d*_a_ are zero, and the mass coefficient *e*_a_ remains l.

The relation between $W_{1\sim 5}$ and W was also constructed by writing it directly into COMSOL6.1 in the form of Equation (6).

### 2.4. Validation Based on the PDE Method

**Figure 2:**
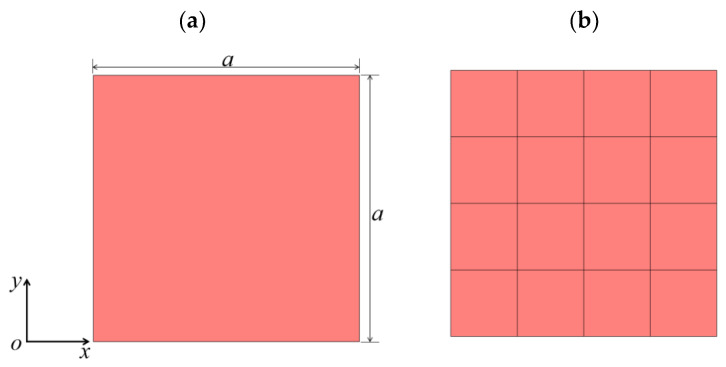
A single material piezoelectric PC plate: (**a**) a schematic diagram of a single cell and (**b**) the finite periodic sequence diagram.

**Figure 3:**
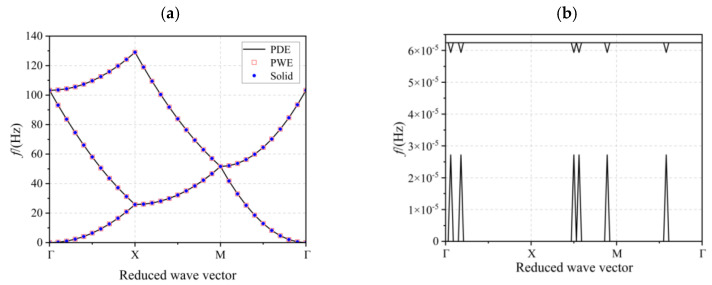
Energy band structure diagram: (**a**) comparison of the energy band structures of the three methods and (**b**) the energy band structure obtained using the reduced-order method in the literature [32].

To verify the correctness of the above method simply and effectively, as shown in Figure 2, we considered replacing the material of the plate structure shown in Figure 1 with an epoxy resin. The lattice constant is still *a*. Based on the above proposed method, the control equations of the plate were solved under the PDE module of COMSOL6.1. The results were compared with those solved using the conventional PWE method and the COMSOL6.1 solid mechanics module. See Figure 3a, from which it is obvious that the energy band structure diagram obtained using the three methods completely overlaps, which fully verifies the correctness of the above methods. Throughout the calculation process, we found that, with consistent mesh delineation, the solving time using the COMSOL6.1 solid mechanics module was 56 s, whereas the solving time using the PDE module was only 13 s, greatly improving calculation efficiency. Compared with the traditional PWE method, this method can solve complex structures more simply and can directly obtain the transmission curves and vibration modes.

It is worth noting that a 2D nanoscale circular hole PC model was also solved in the literature [32] using the PDE module in COMSOL6.1. To transform the equations into the form of the built-in equations of the PDE module in COMSOL6.1, a reduced-order method was also introduced in the literature. We solved the plate structure shown in Figure 2 using this reduced-order method, and the results are shown in Figure 3b. The energy band structure appeared to be disordered. After continuous investigation, we found that when using the reduced-order method in the literature [32] to solve Equation (1), it does not take into account the fact that the material group element parameters are also periodic functions in the space (*x*, *y*), which need to be considered, and as a result, correct results could not be obtained. Therefore, in terms of solving 2D piezoelectric PC structures using the PDE module in COMSOL6.1, the reduced-order approach mentioned in the previous section is essential.

## 3. Numerical Results and Analysis

### 3.1. Energy Band Structure

**Figure 4:**
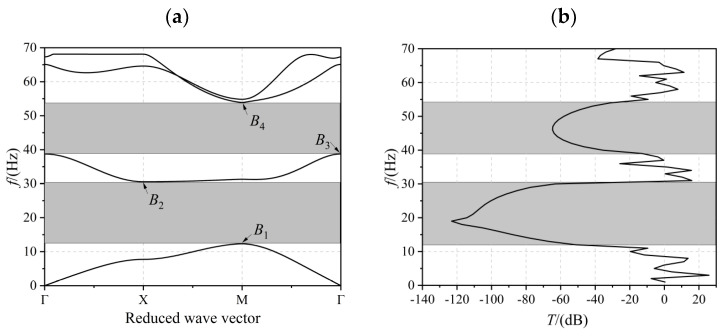
Diagram of the energy band structure (**a**) and transmission profile (**b**) of the piezoelectric PC plate shown in Figure 1.

The energy band structure of the structure shown in Figure 1a is given in Figure 4a, from which it can be seen that two complete bandgaps were opened below 55 Hz (shown in the grey area), with a first-order bandgap bandwidth of 18.25 Hz. The second-order bandgap bandwidth was 15.19 Hz. Figure 4b gives the transmission curve diagram of the finite periodic sequence structure shown in Figure 1b. It can be seen from Figure 4 that the vibration attenuation frequency band and the bandgap range coincide, indicating that the piezoelectric PC board structure has a good vibration and noise reduction effect.

**Figure 5:**
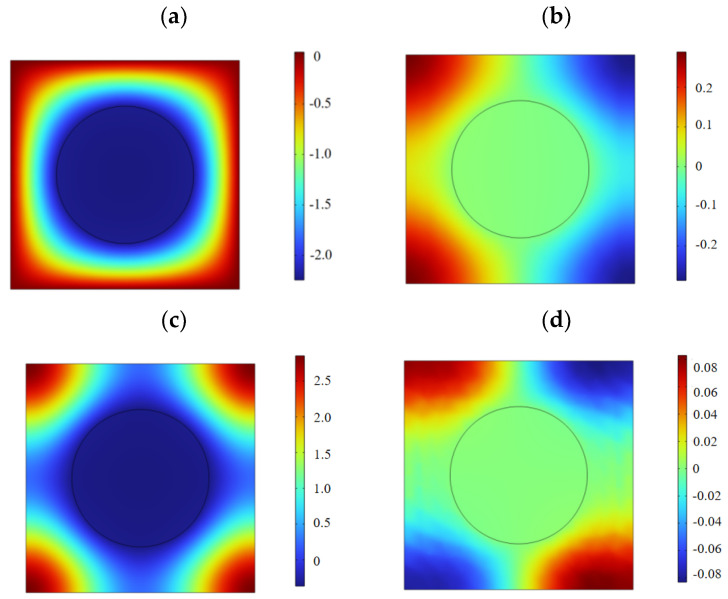
The displacement fields of the eigenmodes labeled in Figure 4: (**a**) *B*_1_, (**b**) *B*_2_, (**c**) *B*_3_, and (**d**) *B*_4_.

The displacement field corresponding to the eigenmode of the critical point in the energy band diagram was extracted to reveal the bandgap characteristics. As shown in Figure 5, for mode *B*_1_, the epoxy resin edge portion did not move, and the portion near the piezoelectric material PZT-4 was concave downwards, presenting a kind of bowl-like movement. *B*_2_ is a PZT-4 material that does not move; the left epoxy resin moves upward and the right epoxy resin moves downward, presenting a kind of origin-symmetric movement. *B*_3_ is the same PZT-4 material that does not move; the epoxy resin part moves upward along the four corners of the movement, presenting a kind of petal movement. *B*_4_ also demonstrates an origin-symmetric movement; the epoxy resin part moves upwards on the upper-left and lower-right and downwards on the lower-left and upper-right.

### 3.2. The Order of Magnitude of Thickness, the Effect of Voltage on the Bandgap, and the Dependence between Them

Piezoelectric materials have received extensive attention from scholars because of their tunable properties, and regulating their properties by changing the voltage is an effective approach for practical engineering applications. Therefore, this study used the above approach to explore the effect of voltage on bandgap. For the structure shown in Figure 1, we investigated its effect on the energy band structure by controlling its thickness and voltage as single variables. For the same absolute value, applying a negative voltage was far better than applying a positive voltage because, in this article, applying a negative voltage was chosen for discussion. The energy band structure maps were calculated for *h* = 1 × 10^−2^, 1 × 10^−3^, and 1 × 10^−4^ m thicknesses, respectively. The first- and second-order bandgaps were selected to study the effect of voltage on the bandgap at different thickness magnitudes.

**Figure 6:**
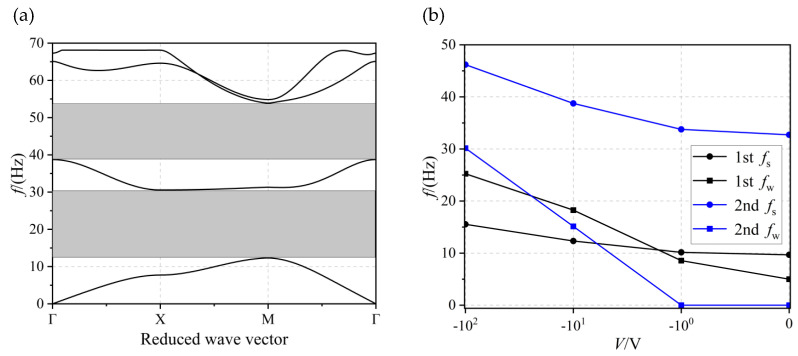
(**a**) The energy band structure diagram and (**b**) the effect of voltage on first- and second-order bandgap when *h* = 1 × 10^−4^ m.

Figure 6a shows the structure of the energy band at a thickness of *h* = 1 × 10^−4^ m and a voltage of *V* = −10 V. At this time, two complete bandgaps were opened (shown in the grey area), from 12.37 to 30.62 Hz (with a bandwidth of 18.25 Hz) and from 38.77 to 53.96 Hz (with a bandwidth of 15.19 Hz), respectively. The effect of voltage on the onset frequency *f*_s_ and the bandgap width *f*_w_ of the first two orders of the bandgap when *h* = 1 × 10^−4^ m is given in Figure 6b, from which it is obvious that the widths of the first two orders of the bandgap were gradually increased with increasing voltage. Furthermore, the onset frequency of the bandgap gradually shifted upwards. The change of the second bandgap from none to all was achieved in the interval from −1 V to −10 V.

**Figure 7:**
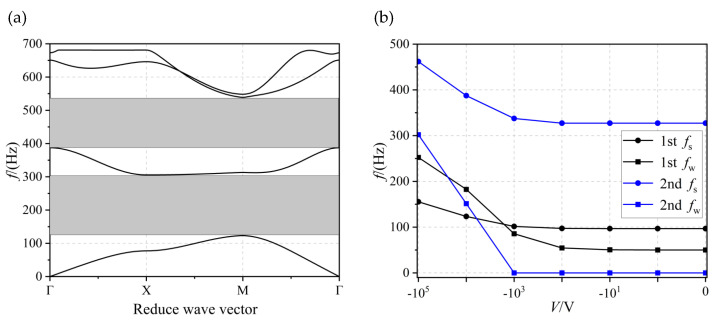
(**a**) the energy band structure diagram and (**b**) the effect of voltage on first- and second-order bandgap when *h* = 1 × 10^−3^ m.

Figure 7a shows the structure of the energy band at a thickness of *h* = 1 × 10^−3^ m and a voltage of *V* = −10^4^ V. It is also possible to open two complete bandgaps (shown in the grey area), 123.73 to 306.2 Hz (with a bandwidth of 182.47 Hz) and 387.72 to 539.65 Hz (with a bandwidth of 151.93 Hz), respectively. Figure 7b shows the effect of voltage on the bandgap at *h* = 1 × 10^−3^ m; however, in the interval from 0 V to −100 V, the voltage does not affect the bandgap. By continuing to increase the voltage, the width of the bandgap became larger with increasing voltage, and the onset frequency *f*_s_ of the bandgap gradually shifted upwards. Within the interval from −10^3^ V to −10^4^ V, the second bandgap could be realized from none to some.

**Figure 8:**
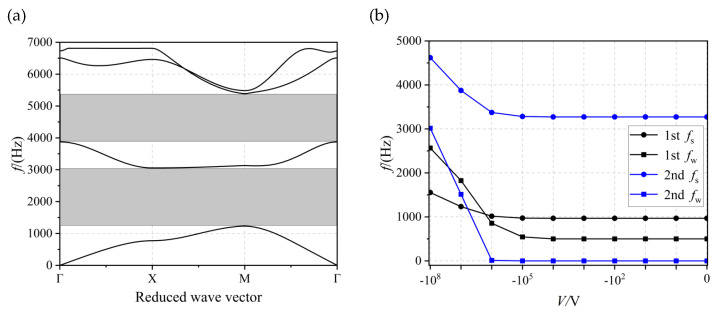
(**a**) The energy band structure diagram and (**b**) the effect of voltage on first- and second-order bandgap when *h* = 1×10^−2^ m.

Figure 8a shows the structure of the energy band at a thickness of h = 1 × 10^−2^ m and a voltage of V = −10^7^ V. Again, two complete bandgaps were opened (shown in the grey area), from 1237.35 to 3062.58 Hz (with a bandwidth of 1825.23 Hz) and from 3877.20 to 5396.5 Hz (with a bandwidth of 1519.3 Hz), respectively. Figure 8b shows the effect of voltage on the bandgap when *h* = 1 × 10^−2^ m. In the interval from 0 V to −10^5^ V, the voltage did not affect the bandgap. As the voltage continued to increase, the bandgap width started to increase and the onset frequency *f*_s_ of the bandgap started to move up. At this point, the voltage had to be within the −10^6^ V to −10^7^ V interval before the second bandgap could be opened.

By comparing and analyzing Figure 6, Figure 7 and Figure 8, the starting frequencies of the first-order bandgap were 12.37 Hz, 123.73 Hz, and 1237.35 Hz, respectively. We found that when the thickness of the piezoelectric PC plate structure was one order of magnitude higher, the band where the bandgap was located also correspondingly increased by one order of magnitude. In addition, there was a relationship between the magnitude of the thickness and the bandwidth of the voltage change. When *h* = 1 × 10^−2^ m, the voltage had to be applied to a magnitude greater than −106 before the first-order bandgap began to change significantly and the second-order bandgap began to open. Such a large magnitude of voltage cannot be applied in practical engineering. When *h* = 1 × 10^−3^ m, applied to an order greater than −10^3^ V, the bandgap width increased dramatically and the second-order bandgap opened up. When *h* = 1.0 × 10^−4^ m, only a conventional voltage was needed to change the first-order bandgap and to open the second-order bandgap. From this, we concluded that when the magnitude of the piezoelectric material thickness was smaller, it was easier to change the bandgap width by applying a voltage, which in turn meets the needs of practical engineering.

Through the above study, when *h* = 1.0 × 10^−4^ m, two complete bandgaps could be opened at ultra-low frequency, and the bandgap width could be changed by applying a conventional voltage to meet the demand for versatility in practical engineering applications.

### 3.3. Effect of Geometrical Parameters on Bandgap

For the structure shown in Figure 1, we controlled its lattice constant *a* and embedded circle radius *r* and thickness *h* as single variables to study their effects on the onset frequency *f*_s_ and bandgap width *f*_w_ of the first- and second-order bandgaps, respectively.

**Figure 9:**
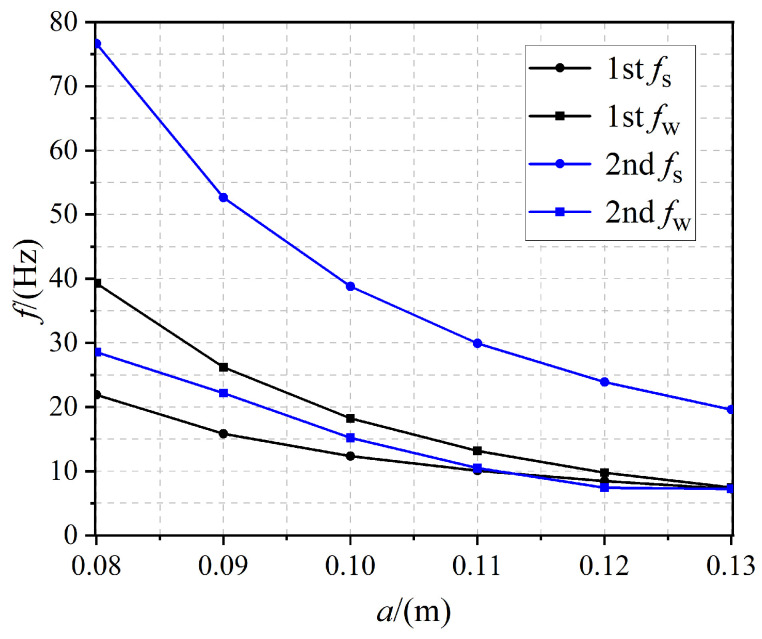
The effect of lattice constant *a* on the first- and second-order bandgap.

As shown in Figure 9, we investigated the effect of lattice constant *a* on the first- and second-order bandgap, and the interval of *a* ranged from 0.08 to 0.13 m. With an increase in *a*, the width of the bandgap of the first two orders decreased gradually, but the band in which the bandgap was located gradually shifted to low frequency and the magnitude of the change was large. It can be seen that the change of lattice constant *a* from 0.08 m to 0.13 m reduced the first-order bandgap width by about 80 percent and the second-order bandgap width was reduced by about 82 percent.

**Figure 10:**
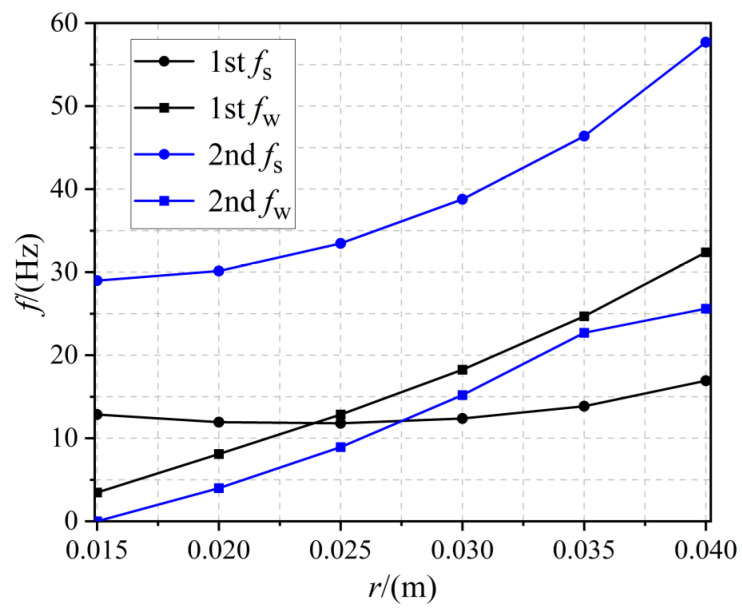
The effect of embedded circle radius *r* on the first- and second-order bandgap.

The effect of the radius *r* of the embedded circle on the bandgap is shown in Figure 10. All the other parameters were consistent with the structure shown in Figure 1. It can be seen that, in the interval from 0.015 to 0.040 m, the first two orders of the bandgap band gradually moved upwards with increasing *r*, and the width of the bandgap also increased. Therefore, we can control the bandgap width and the frequency band where it is located by adjusting the radius *r* (the proportion of piezoelectric material) of the embedded circle. When the radius *r* changed from 0.015 m to 0.04 m, the first-order bandgap width increased by a factor of about 8.3 and the second-order bandgap width remained unchanged.

**Figure 11:**
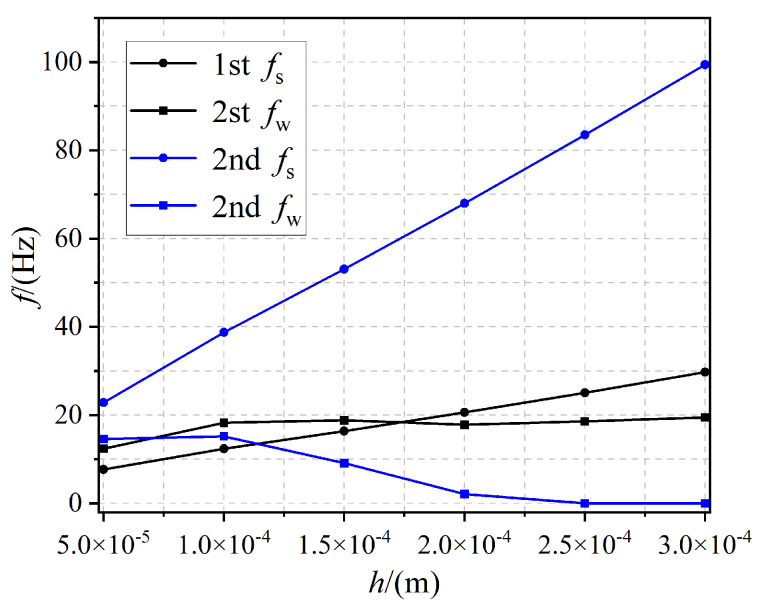
The effect of thickness *h* on the first- and second-order bandgap.

The effect of thickness *h* on the onset frequency fs and bandgap width *f*_w_ of the first two orders of the bandgap is shown in Figure 11. The interval segment of *h* was 5 × 10^−5^ to 3 × 10^−4^ m. As *h* increased, the first two starting frequencies *f*_s_ gradually increased, and the first-order bandgap width increasef in the interval of thickness *h* of 5 × 10^−5^ to 1 × 10^−4^ m, which varied slightly in the interval of 1 × 10^−4^ to 3 × 10^−4^ m. The second-order bandgap was unchanged in the interval of thickness *h* of 5 × 10^−5^ to 1 × 10^−4^ m, decreased substantially in the interval of 1 × 10^−4^ to 2.5 × 10^−4^ m, and the bandgap width was unchanged in the interval of 2.5 × 10^−4^ to 3 × 10^−4^ m. Overall, the variation of thickness *h* in the interval from 5 × 10^−5^ to 3 × 10^−4^ m had little effect on the bandgap width. When the thickness *h* changed from 5 × 10^−5^ to 3 × 10^−4^, the first bandgap width increased by 25 percent and the second bandgap faded away.

## 4. Conclusions

This paper constructs a piezoelectric PC plate structural model for marine applications by embedding PZT-4 in epoxy resin and arranging it periodically in the *xy* direction. By combining the Fourier series expansion theory and finite element theory, a new method to solve the control equations of a two-dimensional piezoelectric PC structure was proposed using the PDE module in COMSOL6.1. The correctness of the method was verified by comparing the results with those solved by the traditional PWE method and the solid mechanics module. Based on this method, the relationship between the thickness order of magnitude, the band order of magnitude where the bandgap is located, and the effect of voltage on the bandgap was explored, and the effects of dimensional parameters on the bandgap characteristics was investigated. The following conclusions were drawn:

1. The simulation results obtained using the method proposed in this paper are in full agreement with those solved using the PWE method and the solid mechanics module in COMSOL6.1, which verifies the feasibility of the method. During the calculations, it was found that the computational speed of the method substantially increased compared to solving it using the solid mechanics module. Compared with the PWE method, this method directly obtained the transmission curve map and the vibration modal map.
2. As the thickness unit of the piezoelectric PC sheet structure decreased by one order of magnitude, the frequency band in which the bandgap was located correspondingly decreased by one order of magnitude, and the order of magnitude of the voltage that needed to be applied to change this bandgap decreased. The adhered piezoelectric PC sheet made it easier to control the bandgap by changing the voltage, providing a new reference for engineering applications.
3. In practice, the bandgap can be modulated by varying its lattice constant *a*, the radius *r*, and the thickness *h* of the embedded circle.

In this study, the bandgap characteristics of a piezoelectric PC plate were investigated based on the above method. However, the current study remains limited to one and two dimensions. Scholars need to continuously explore how to apply this method to three dimensions.

## Figures and Tables

**Table 1:** Calculations for different materials.

Category	PZT-4	Epoxy
m	$\frac{h^{3}}{12}\left(\tilde{c_{11}}+\frac{{\tilde{e_{31}}}^{2}}{{\tilde{\kappa_{33}}}^{2}}\right)$	$\frac{h^{3}}{12}\frac{E}{1-\mu^{2}}$
n	$\frac{h^{3}}{12}\left(\tilde{c_{12}}+\frac{{\tilde{e_{31}}}^{2}}{{\tilde{\kappa_{33}}}^{2}}\right)$	$\frac{h^{3}}{12}\frac{E\mu }{1-\mu^{2}}$
p	$\frac{h^{3}}{6}\tilde{c_{66}}$	$\frac{h^{3}}{12}\frac{E}{1+\mu }$
q	$\tilde{e_{31}}\cdot$ *V*	0
l	$\rho_{1}\cdot h$	$\rho_{2}\cdot h$

**Table 2:** Material parameters covered in the text.

Parameters of PZT-4	Value	Parameters of Epoxy	Value
$\rho_{1}$/kg·m^−3^	7500	$\rho_{2}$/kg·m^−3^	1180
$c_{11}$/Gpa	132	*E*/Gpa	4.35
$c_{12}$/Gpa	71	μ	0.368
$c_{13}$/Gpa	73		
$c_{33}$/Gpa	115		
$c_{66}$/Gpa	30.5		
$e_{31}$/C·m^−2^	−4.1		
$e_{33}$/C·m^−2^	14.1		
$\kappa_{33}$/CV^−1^m^−1^	7.124 × 10^−9^		

**Table 3:** Implementing Equation (13) in COMSOL (using only the left of the equal sign as an example of the first term).

Parameters	Items
*e*_a_	l
*d*_a_	0
Γ	$ik_{x}W_{1Kx}+W_{1Kxx}$
*f*	$-k_{x}^{2}W_{1K}+ik_{x}W_{1Kx}$

**Table 4:** Implementing Equation (8) in COMSOL.

Parameters	Items
*e*_a_	0
*d*_a_	0
Γ	$m\left(ik_{x}W_{Kx}+W_{Kxx}\right)$
*f*	$W_{1K}-{m(-k}_{x}^{2}W_{K}+ik_{x}W_{Kx}$)

## Data Availability

Data are contained within the article.
